# Cellular and metabolic mechanisms of nutrient actions in immune function

**DOI:** 10.1038/s41387-021-00162-3

**Published:** 2021-06-23

**Authors:** Philip Newsholme

**Affiliations:** Curtin Medical School Faculty of Health Sciences and Curtin Health Innovation Research Institute, Curtin University, Perth, WA 6845 Australia

**Keywords:** Inflammatory diseases, Metabolism

## Abstract

Various nutrients can change cell structure, cellular metabolism, and cell function which is particularly important for cells of the immune system as nutrient availability is associated with the activation and function of diverse immune subsets. The most important nutrients for immune cell function and fate appear to be glucose, amino acids, fatty acids, and vitamin D. This perspective will describe recently published information describing the mechanism of action of prominent nutritional intervention agents where evidence exists as to their action and potency.

## Introduction

The immune system protects the host against infection from pathological microorganisms and provides constant surveillance for malignant cells that arise over a lifetime. The immune system is able to develop appropriate tolerance to self-proteins, circulating macromolecules, self-cells, and tissues, and to harmless environmental molecules [1].

Individual heterogeneity regarding the intensity of immunological responses exists, largely dependent on genetics, environment, lifestyle, nutrition, and the interaction of these factors. Nutritional immunology is a field of immunology that describes the influence of nutrition on the immune system, antiviral activity, and associated protective functions [2]. Deficiency in macronutrients and/or micronutrients causes impairment of immune function, which usually can be reversed by nutrient repletion. A poor diet composition which is common in the elderly population, and excess calorie consumption are a problem. Nutritional deficiency or insufficiency must be corrected to ensure normal immune functions including maintaining tolerance. While recognition exists for the importance of various nutrients, the mechanism of action is often not considered by non-molecular scientists.

This perspective will describe recently published reports describing the mechanism of action of prominent nutritional intervention agents where evidence exists as to their action and potency (glutamine and arginine, fatty acids such as n-3 polyunsaturated fatty acids or medium-chain fatty acids, micronutrients e.g., vitamin D). This perspective has concentrated on describing the molecular and/or metabolic basis of their immunological effect.

## Amino acids and immune function

### Glutamine

Glutamine is the most abundant amino acid in the blood and in the free amino acid pool in various tissues [3]. The concentration of glutamine in the blood is 2× to 100× greater than other amino acids, at ~0.7 mM. Skeletal muscle is quantitatively the most important source of glutamine, it is released into circulation from the muscle. The are several major consumers of glutamine, namely kidney, liver, gut, and cells of the immune system (see Figures 2, 3 in ref. [3]). The blood glutamine concentration can fall by up to 50% in conditions of sepsis, injury, burns, and post surgery, while at the same time, skeletal muscle glutamine concentration may fall by up to 50%. The demand for glutamine will be much higher in conditions of trauma, impacting on skeletal muscle glutamine synthesis and export, which will both be elevated in such conditions.

It is widely accepted that immune cell intermediary metabolism is critical to cell function. Glucose is mainly converted into lactate (glycolysis), whereas glutamine is converted into glutamate, aspartate, and alanine by undergoing partial oxidation in the TCA cycle to CO_2_, aspartate, alanine, and lactate in a process called glutaminolysis (see Figure 3 in ref. [3]). Furthermore, through the pentose phosphate pathway, cells can produce ribose-5-phosphate (a five-carbon sugar), which is a precursor for the pentose sugars required for the synthesis of RNA and DNA, as well as glycerol-3-phosphate for phospholipid synthesis. The degradation of glutamine, and thus formation of carbamoylphosphate using the amide nitrogen of glutamine and also formation of aspartate through glutaminolysis, leads to the synthesis of pyrimidines required for DNA and RNA synthesis. The expression of several genes in immune system cells is largely dependent on glutamine availability and consumption [3]. For example, the proliferation of immune cells occurs through activation of key enzymes and dependent on glutamine availability, such as the ERK and JNK kinases. These kinases may activate key transcription factors, such as JNK and AP-1, and thus leading to the transcription of cell proliferation-related genes. Glutamine is also required for the expression of key lymphocyte cell surface markers, such as CD25, CD45RO, and CD71, and the production of cytokines, such as interferon gamma (IFN-γ), TNF-α, and IL-6 [4–7]. Thus, glutamine can act as an energy substrate for leukocytes, and a biosynthetic precursor and a transcription factor activator thus playing a key role in cell proliferation, tissue repair, and mechanisms associated with pathogen recognition [8].

Different populations of macrophages have now been identified, including M1 and M2 macrophages [9–11]. M1-like macrophages are responsible for secreting pro-inflammatory cytokines and lipid mediators and are involved in tissue degradation and T-cell activation. M2 macrophages exert different functions, such as contribution to tissue repair and the secretion of anti-inflammatory cytokines and lipid mediators. Treatment of macrophages with LPS in vitro promotes a switch from glucose-dependent oxidative phosphorylation to aerobic glycolysis—typical of the Warburg effect [12]. Pyruvate kinase M2 regulates hypoxia-inducible factor 1-alpha (Hif-1α) activity and IL-1β expression, thus being a key regulator of the Warburg effect in LPS-activated macrophages [13]. Due to this mechanism, M1 macrophages exhibit a rapid increase in ATP formation that is required for the host-defense response [14]. M1 macrophages (treated with LPS) have two points of substrate flux deviation with regard to the TCA cycle unlike M2 macrophages, one occurring at the isocitrate dehydrogenase step and another post succinate formation. As a result, there is an accumulation of TCA cycle intermediates (e.g., succinate, α-ketoglutarate, citrate, and itaconate) that impacts the activation of LPS stimulated macrophages [15, 16]. Itaconate, a recently discovered metabolic regulator in macrophages has anti-inflammatory properties through activation of nuclear factor erythroid 2-related factor 2 (Nrf2) via Kelch-like ECH-associated protein 1 (KEAP1) alkylation [8]. In addition, Liu et al. [17] reported α-ketoglutarate, generated through glutaminolysis, promotes M2 macrophage differentiation. Macrophage metabolism varies with specific tissue microenvironment, and this is required for macrophage differentiation and function. For example, the peritoneum is rich in glutamate, a product of glutamine catabolism, that is used by resident peritoneal macrophages to induce specific metabolic changes required for microbial sensing and removal [18].

Glutamine metabolism and supply of metabolic intermediates have been proposed to be essential to the antiviral activity of the immune system, although many of the mechanisms are as yet unknown [19].

### Arginine

The plasma concentration of l-arginine is 0.077 mM, approximately tenfold less than glutamine. Arginine is particularly important in the liver, where it is an intermediate in the urea cycle, thus is an intermediate in amino acid catabolism. Arginine is also very important in a number of immune cells, as a substrate for nitric oxide synthase (NOS), thus is a substrate for the production of nitric oxide, a potent immunoregulatory mediator that is cytotoxic to tumor cells and many microorganisms. Macrophages can produce relatively large amounts of nitric oxide, especially the M1 inflammatory macrophage, via increased concentrations of the enzyme inducible NOS (iNOS). The activated macrophage can actually release the enzyme arginase to deplete local concentrations of arginine (indeed arginine is required for tumor growth). It has been reported that mouse peritoneal resident and Bacillus Calmette–Guerin (BCG)-activated macrophages and human monocytes are capable of utilizing glutamine at high rates, contain sufficient activity of the enzymes required to convert glutamine to citrulline (and subsequently citrulline to arginine via enzymes of a partial urea cycle) to account for observed rates of nitrite synthesis (derived from NO) in the absence of extracellular l-arginine, and will release nitrite when exposed to intermediates of the proposed glutamine --> arginine pathway [20]. Thus the M1 macrophage can release arginase to deplete local concentrations of arginine but ensures it has sufficient intracellular arginine via biosynthesis from glutamine. In humans, myeloid-derived suppressor cells store ARG1 in granules and release it to the extracellular milieu. It leads to the depletion of extracellular l-arg and indeed may play a role in the suppression of the antitumor response [21].

## Lipids and immune function

Fatty acids (FAs) play several essential roles in the homeostasis and structure of the cell and various tissues and organs. FAs are the main components of all biological membranes and are built into sphingolipids, phospholipids, glycolipids, and lipoproteins. They are also the major source of energy stored in the triacylglycerols. In addition, various metabolites of FAs serve as essential intracellular and extracellular lipid mediators and hormones. Therefore, FAs have many possibilities to modulate immune cell function by influencing their structure, metabolism, and function, acting through surface proteins (G-protein-coupled receptors; GPRs), nuclear receptors, or membrane transporters [22]. Much has been published on the impact of lipids on immune cell function [2]. Saturated fatty acids have little impact on humoral or cell-mediated immune function but in cell culture, animal models, and human epidemiological studies some saturated fatty acids promote inflammation. However, cell culture and animal model studies have reported that marine n-3 PUFAs (EPA and docosahexaenoic acid [DHA]) can suppress both inflammation and cell-mediated immune responses, acting through a variety of mechanisms which may include alterations to membrane structure, signaling processes, gene expression, and/or production of eicosanoids.

Many viruses, including coronaviruses, are enveloped viruses with a nucleocapsid mainly made of phosphorylated nucleocapsid protein embedded inside a phospholipid bilayer envelope. This envelope plays an important role in virus assembly and release from the host cell, and it is critical for viral pathogenesis. The viral surface consists of the spike protein of the virus, which is essential for the attachment to the host cell receptor and for initiating infection. Given the indispensable nature of lipids in multiple stages of viral replication, lipid synthesis and metabolism in host cells have been recognized as essential for the assembly of coronaviruses. Bioactive lipids with antiviral properties include certain long-chain monounsaturated fatty acids (MUFAs) and medium-chain fatty acids (MCFAs), specifically lauric acid (LA), the primary fatty acid of coconut oil. It is a saturated MCFA with potent antimicrobial properties [23]. Long-chain unsaturated fatty acids (such as oleic acid, a major constituent of olive oil) are also active against enveloped viruses such as coronavirus. Monoglycerides of these fatty acids also show significant antiviral activity. Antiviral fatty acids impact the viral envelope, causing leakage and at higher concentrations can cause a complete disintegration of the viral particles including the viral envelope. Also, MCFAs can prevent the binding of viral proteins to the host cell membrane as described for infectious *Vesicular stomatitis virus* [23].

## Vitamin D

The active form of vitamin D (1,25-dihydroxy vitamin D3) is usually abbreviated as vitamin D. Vitamin D receptors have been identified in most immune cells [24]. Macrophages can synthesize the active form of vitamin D from its circulatory precursor. Vitamin D can induce macrophages to synthesize antimicrobial peptides, such as cathelicidin. There is evidence to suggest immune defects in vitamin D-deficient patients and experimental animals leading to susceptibility to infections. However, there is a large body of literature supporting the immunosuppressive role of vitamin D and related analogs. It is probable that under physiological conditions, vitamin D may promote appropriate immune responses but it is required in the prevention of autoimmunity or suppressing low-grade inflammation [24, 25]. Vitamin D acts by binding to its receptor and regulating gene expression in target cells. Its effects include the promotion of phagocytosis, superoxide synthesis, and bacterial killing, but it is also reported to inhibit T-cell proliferation, production of Th1 cytokines, and B-cell antibody production. The inhibition of Th1-type immune activity, which underlies many autoimmune conditions, by vitamin D, is thought to be key to the action of the vitamin [24].

A recent study reported for the first time the potential influence of vitamin D status on bioenergetics and metabolism in freshly isolated peripheral blood mononuclear cells. The data indicated a close relationship between Vitamin D levels and immune cell bioenergetic responses. Low vitamin D levels were clearly associated with a pattern consistent with increased oxidative metabolism and inflammatory activation that was reflected in altered bioenergetics of PBMCs [25].

## Essential molecular and metabolic research areas for future studies

- Studies on the effects of nutrient combinations, whole foods, and diets on human immune cell metabolism and function, in vivo and ex vivo
- Nutritional supplementation and potential prevention or slowing of immunosenescence by altering immune cell metabolism and function
- Investigation of the role of obesity in altering immune cell metabolism and function in humans, including antiviral activity
- The role of the gut microbiota in shaping host immune maturation, metabolism, and functional responses in animals and humans including antiviral activity
- Determination of the role of glutamine metabolism and supply of glutamine derived metabolic intermediates in the essential antiviral activity of the immune system (adapted from ref. [24]).

## Abbreviations

ERK: Extracellular Signal-Regulated Kinases
JNK: c-Jun N-terminal Kinase
AP-1: Activator protein 1
CD: Cluster of Differentiation
TCA cycle: Tricarboxylic acid cycle
LPS: Lipopolysaccharides
EPA: Eicosapentaenoic acid
